# Emphasizing Science: Black Pastors Encourage Awareness of Medical Information about COVID-19 Vaccine

**DOI:** 10.5539/gjhs.v18n2p27

**Published:** 2026-03-20

**Authors:** DaKysha Moore, Nicole Caviness-Ashe, Lisa Mansfield, Elijah O. Onsomu

**Affiliations:** 1 Department of Communication Sciences and Disorders, North Carolina Agricultural and Technical State University, 1601 E Market Street, Greensboro, NC 27411, USA.; 2 Cancer Prevention and Control Training Program, Heersink School of Medicine, The University of Alabama at Birmingham, 1720 2^nd^ Ave S, Birmingham, AL 35233, USA.; 3 School of Nursing, The University of North Carolina at Chapel Hill, Campus Box #7460, Chapel Hill, NC 27599, USA.; 4 Division of Nursing, Winston-Salem State University, 601 S. Martin Luther King Jr. Dr., Winston-Salem, NC 27110, USA.

**Keywords:** Church Leadership, COVID-19, Health Communication, Immunization, South Carolina

## Abstract

Church leaders have used scripture to encourage congregants to reflect on their health choices. Still, this study focuses on Black pastors who used other approaches to prompt awareness about COVID-19 and vaccination. The purpose of the study is to explore how Black posters use medical information and scripture to discuss COVID-19 vaccination within the communities they serve. This study uses a phenomenological design; ten Black pastors in South Carolina were recruited through snowball sampling to learn about their information dissemination practices during the COVID-19 pandemic. The study describes their rhetoric, foregrounding medical facts and supporting them with biblical passages. The pastors who participated in semi-structured interviews presented scientific information to the church community and encouraged members to “follow the science.” Two central themes emerged from the study: 1) Stand by the science to educate the Black community about COVID-19 vaccines, and 2) Balancing science and scripture to reinforce positive COVID-19 vaccine messaging. The study’s implication demonstrates how Black pastors are instrumental in providing a narrative of factual health information in a spiritual setting.

## 1. Introduction

Throughout the COVID-19 pandemic, several strategies were used to promote vaccine uptake among the Black population. These strategies include engaging community leaders to raise awareness of safety practices during the pandemic. One addressed reason for hesitancy, acknowledging the role of mistrust stemming from historical medical abuses and mistreatment (Roat et al., 2023; Savoia et al., 2024). Public health officials began collaborating with trusted community messengers, such as Black pastors. With authority and social influence in their respective communities, they were essential for disseminating reliable, consistent COVID-19 vaccine information, increasing access to vaccine distribution, and promoting vaccine uptake in Black communities, especially at the beginning of the pandemic, when misinformation was widespread (Skafle et al., 2022).

Although Black medical providers and pastors worked together, developing targeted messages to increase COVID-19 vaccination among underserved and minoritized groups, especially Black Americans (Dada et al., 2022), disparities in vaccine uptake persist. As of July 2022, for most reporting states, people who identified as Black had a lower rate of COVID-19 vaccination compared to Asians, Whites, and Hispanics (Ndugga et al., 2022). Across South Carolina, Black Americans and White Americans showed disparate percentages of cases and mortality rates (Monk et al., 2020; Yang et al., 2021). In the early phases of the pandemic, the elderly, men, and some minoritized groups, especially Black communities, had higher rates of hospitalization and mortality (Yang et al., 2021). Fewer than 50 percent of residents were fully vaccinated at the time of data collection, July 2021 (South Carolina Office of the Governor, 2021).

Because of disparities in overall health outcomes among Black Americans, trusted messengers were needed to promote COVID-19 vaccination. Reasons for mistrust include historical factors, the Tuskegee Experiment, and more recent events, such as civil unrest after the death of George Floyd (Bunch, 2021; Mansfield et al., 2022). As such this paper describes the strategies pastors used to promote COVID-19 vaccine awareness and knowledge in predominantly Black churches in South Carolina while balancing the dissemination of scientific information in a religious setting.

Pastors often emphasize holistic care when addressing health-related concerns, and research has examined how Bible-based teaching and scripture are incorporated into health promotion among Black congregants. Gutierrez et al. (2014) showed that using biblical sources to educate Black and Latino New Yorkers about healthy behaviors led to healthier eating and increased physical activity. Incorporating scripture into diabetes education was also found to be effective (Krisberg, 2012). In a study by Tettey et al. (2016), Black participants in faith-based communities reported that biblical teachings helped align scientific information with their perspectives, thereby increasing its credibility. Moreover, Tettey et al. (2017), in the same population, used biblical teaching to raise awareness of cardiovascular disease; they achieved this goal, and participants overall lowered their blood pressure.

Other studies have reviewed how faith-based partnerships can increase awareness about various diseases and chronic illnesses (DeHaven et al., 2004; Williams et al., 2016). Researchers have collaborated with pastors to assess the impact of interventions aimed at improving health outcomes among Black congregants (Gross et al., 2018; Williams & Cousin, 2021). Pastors have facilitated study interventions to increase physical activity (Thompson, Berry, & Hu, 2013; Wilcox et al., 2007) and reduce the risk of heart disease (Frank & Gribbs, 2008). Religious/spirituality-based programs in the Black community have helped participants manage breast cancer (Hardy & Bugella, 2019; Holt & Klem, 2005; Holt et al., 2008) and prostate cancer (Drake et al., 2010; Husaini et al., 2008; Saunders et al., 2015).

Since the COVID pandemic, researchers have assessed the role of faith-based organizations in promoting vaccine uptake. Lack of trust has been a catalyst for vaccine hesitancy in the Black community, so Black healthcare providers have collaborated with Black pastors, viewed by their congregations as trusted health messengers (Dada et al., 2022). Privor-Dumma and King (2020) also stress the importance of Black pastors in building trust between Black populations and the healthcare system to increase vaccine uptake.

As part of a larger research project, Moore et al. (2022, 2025) focused on the communication strategies Black pastors in South Carolina used to ensure their church members and surrounding communities were informed about the COVID-19 vaccine. Specifically, they discussed their own COVID-19 vaccine experiences to encourage congregants to feel more comfortable about it. Here, we assess their use of a scientific approach, rather than biblical messaging.

## 2. Methodology

We used a phenomenological design (Creswell, 2013) to elucidate participants’ experiences of COVID-19 vaccine messaging used by Black pastors of predominantly Black churches in South Carolina. The design enabled the researchers to use “intentional analysis” (Wertz, 2023), which enabled the pastors to reflect on whether scriptures were used to disseminate information about COVID-19 vaccinations. An interview guide adapted from a previous study exploring Black pastors’ dissemination of HIV information (Moore et al., 2012) posed 11 questions about the communication channels they used to disseminate COVID-19 vaccine information and to encourage vaccination. The University IRB Institutional Review Board approved the study.

The researchers used snowball sampling to contact potential study participants, assess their eligibility, and obtain their consent. Snowball sampling was essential for recruiting the pastors. The recruitment strategy is often used and considered an advantage when researchers seek to recruit participants who are difficult to access and have no or minimal budget to conduct the research study. Sharma (2017) notes an advantage of snowball sampling is that it allows researchers to contact a target group that may not have a list of names. Even though the researchers could find a list, many pastors were not in office because of the pandemic. A disadvantage of using snowball sampling is the lack of participants from diverse backgrounds, perspectives or both (Ting et al., 2025). There is an acknowledgement that the use of snowball sampling could present issues of validity. As participants are not randomly selected, they may have similar networks. However, this sampling technique is beneficial when recruiting a difficult population to sample (Atkinson & Flint, 2001). According to Ting et al. (2025), there can be reduced control over the sample size and audience when using snowball sampling which can result in over or underestimation of a particular group. This could also reduce validity and generalizability of the findings. For this study, the researchers chose to use snowball sampling because it was difficult to contact pastors during the pandemic. Moreover, the researchers focused on a specific population. Inclusion criteria were 1) at least 18 years of age at enrollment and 2) pastors of churches in South Carolina that mainly serve Black congregants.

In July 2021, one researcher conducted semi-structured interviews via telephone. They lasted 15 to 25 minutes. Using a single researcher ensured consistency and established a rapport with the participants, essential components of qualitative interviews (DeJonckheere & Vaughn, 2019). All interviews were audio recorded and transcribed using Otter.ai. The transcripts were reviewed to ensure accuracy.

A codebook was developed using QDA Miner Lite software (Provalis, Montreal, Canada). The codes were based on the interview guide questions. There was a focus on specific texts related to the interview question, “if scripture,” used to discuss COVID-19 vaccination. Transcripts were analyzed using thematic analysis to organize data on experiences (Braun & Clarke, 2012). Based on the text, the information was coded using specific colors and keywords related to the scripture topic.

## 3. Results

A total of 10 pastors completed the interviews. Five served Baptist churches and five African Methodist Episcopal churches, most in urban regions of South Carolina. The pastors were both men and women. Two themes emerged describing vaccine messaging used to disseminate COVID-19 vaccine information among Black pastors.

### 3.1 Stand by the Science to Educate the Black Community about COVID-19 Vaccines

Pastors reported that they did not rely on scripture when disseminating information about the vaccine. One stated, “*I’ve mentioned it in sermon, but I have to admit I have not used Sunday morning platform to just talk about COVID in the Scriptures*” (Pastor 2, AME). Another explained that using specific passages was not a common practice. A few pastors discussed the vaccine, not during services, but during the announcements, which might include some scripture. “*During our morning worship service, we have a pastoral observation period where I do any announcements or highlight anything that I feel necessary or feel really important*.” (Pastor 3, Baptist)

The pastors used scientific data to disseminate information across their respective church communities. They stressed the importance of following guidelines from government health agencies, such as the Centers for Disease Control and Prevention (CDC) and local health departments, to provide accurate information about COVID-19 vaccines and address vaccine misinformation in Black communities.

“There is a lot of misinformation out there, so I just believe that the church really needs to be able to look at the science and realize that God works through the science.”

(Pastor 10, Baptist)

As a trusted member of their community, one pastor shared their risk perception of COVID-19 on one’s health to encourage church members to receive the COVID-19 vaccination, “*Once you see the deaths, and once you go to the funerals with your mask on, and someone is dead prematurely because of COVID, it should inspire you to trust the science that we use every day for everything else*.” (Pastor 10, Baptist)

The pastors discussed how they use illustrations and data to inform the congregation. They stressed the importance of not only taking good care of their own health but also helping protect the people around them. “*The plague will not be lifted until God says remove it from the Earth. We can do our part as it relates to mitigation and trying to bring about some level of herd immunity. We do trust the science, so there is no scripture. We use scientific data and research*.” (Pastor 7, AME)

The church leaders discussed how they also encourage their membership to speak with and listen to medical professionals and government officials who are experts in their fields.

“We have covered it since the vaccination was approved by the FDA. We have encouraged the members to give it a strong consideration of getting vaccinated in consultation with their personal physician. We have used all of our avenues of communication with email, and we do a weekly post phone call that reaches all members, and we announce it during our virtual workshop services.”

(Pastor 1, Baptist)

“We continue to monitor our local or state government—the number of cases, the number of deaths. We break it down by population—old, youth, and we break it down by the townships that are around us.”

(Pastor 8, Baptist)

### 3.2 Balancing Science and Scripture to Reinforce Positive COVID-19 Vaccine Messaging

Most pastors did not use specific passages from scripture to arouse interest in, or convince people to take, the vaccine. However, they discussed attempting to help the church community perceive a possible connection between spiritual beliefs and the use of the vaccine.

“Occasionally, I will mention that the body is the temple of the Holy Spirit, and we are obligated to be good stewards of our bodies.”

(Pastor 1, Baptist)

“We present the facts, and yes, we use scripture to let them know that God wishes above all things that we should not only prosper but that we should have good health.”

(Pastor 8, Baptist)

“One thing we stress mostly—[The] Word does say the Lord wants us to prosper and be in good health…. We don’t want to just come to church and sing and shout. He wants us to be a whole person singing and shouting and taking care of our health. We can’t just sing and shout and not take care of our health. And so, you want to safely worship. So, we want to get our vaccinations so that one day these masks will come off and all the social distancing can stop. But we thank God that He is never far from us.”

(Pastor 6, AME)

Pastors were intentional about avoiding the overuse of scripture and instead balancing it with scientific data. Moreover, they stressed the importance of preventing tones that could be perceived as judgmental or harsh.

“No, I do not try to make it so religious to the extent of trying to apply scripture to it. We use the science, and we use the science to drive through faith—we take it in the name of Jesus. Recognizing that ultimately God heals.”

(Pastor 7, AME)

“You have to find ways because you can’t just tell people “Come get your shots” or judge them. You really don’t want to stand in judgment with people who decide not to get it. But at the same time, you want to encourage them to get it.”

(Pastor 10, Baptist)

The pastors expressed the need to remain sensitive to their congregants’ hesitancy to be vaccinated.

“With the previous experiments, among our people…people still just resistant to government interference, I guess you would say, with vaccinating us as African Americans.”

(Pastor 4, Baptist)

“We use scripture to encourage, and I am not one of those who want to try to make you do something. I want to bring you to the point where you make your own decision. We want you to make your own decision, but we want you to see the facts.”

(Pastor 8, Baptist)

As one pastor suggests, it is essential to make connections between the vaccine and other health issues with which the church is more familiar, such as breast and prostate cancer. Such links can improve understanding of health concerns surrounding COVID-19 and the importance of vaccination. “*I believe that every house of worship needs to find ways to explain to people that we are living because God has blessed the mind and hand of the scientist to heal in multitude rather than one person as a time*.” (Pastor 10, Baptist)

## 4. Discussion

Two central themes developed from interviews with pastors of churches that primarily serve Black residents of South Carolina including standing by the science to educate the Black community about COVID-19 vaccines and balancing science and scripture to reinforce positive COVID-19 vaccine messaging. In interviews regarding the decision to take the vaccine, the pastors focused on scientific information. They understand their pivotal role in discussing health issues as trusted members of the community (Dankwah et al., 2024; Harmon et al., 2018) in the face of past medical mistreatment based on bias (Jaiswal & Halkitis, 2019; Matthews et al., 2002; McDonnell & Idler, 2020; Nguyen et al., 2021). Many church members rely more on information from their pastors than from medical providers and media outlets (Wright & Reed, 2024). Historically, Black pastors have been leaders in areas ranging from social activism to counseling (Cohall & Cooper, 2010; Neighbors, Musick, & Williams, 1998).

Their status has been crucial in saving lives during the pandemic. Studies confirm that medical mistrust accounts for lower vaccination rates among Black people during the initial phase (Dong et al., 2022; McFadden et al., 2022). The interviews suggest that the pastors who took part in this study decided to rely on scientific data, to reinforce health messaging with scriptural references, and to deliver it with care and intentionality, adjusting their tone and the information presented to the needs of their church communities. Throughout the interviews, the pastors recognized their responsibility. Their parishioners perceive them as reliable sources of information, which gives them a cultural advantage over outsiders, however credentialed, in communicating health information. In turn, the pastors work to provide credible, evidence-based information from local, state, or federal health agencies. Privor-Dumm and King (2020) discuss the importance of similar partnerships between Black pastors and outside agencies.

Previous health interventions in the Black church relied on scripture (Tettey et al. 2016; 2017). However, in this study, the pastors did not emphasize the Bible to convey the importance of being immunized. They did not want church members to feel pressured by faith into reluctant compliance. Similarly, Holt et al. (2009) found that when pastors deliver information about colorectal cancer screening, they should avoid preaching. As in the current study, the pastors thought it best not to be judgmental, but to use the Bible to enhance health messages.

## 5. Conclusions and implications

This study supports the idea that Black pastors use specific communication strategies when engaging with their communities about health decisions. The pastors were conscious of how church members perceived their delivery and diction, and they used scriptural references to enhance the importance of taking care of one’s body and health. However, they focused on ensuring congregation members had scientific information from credible sources to make the best decisions about the vaccine. Additional studies are needed to understand and advance the benefits and implications of Black pastors working with health professionals to develop effective communication practices for disseminating new vaccination guidelines and regulations. These strategies may also help develop interventions to mitigate health disparities that disproportionately affect the Black community.

## Data Availability

The data that support the findings of this study are available on request.
